# 

**Published:** 1917-06

**Authors:** 


### CONTENTS.

ORIGINAL COMMUNICATIONS.................... 65

SELECTIONS AND ABSTRACTS.................... 69

BOOK REVIEWS.................................... 96

